# Ethical analysis of the new proposed mental health legislation in England and Wales

**DOI:** 10.1186/1747-5341-2-5

**Published:** 2007-04-12

**Authors:** Peter Lepping

**Affiliations:** 1 North East Wales NHS Trust, Wrexham University of Wales Academic Unit, Wrexham, Wales UK

## Abstract

This paper ethically analyses arising out the proposed changes to the Mental Health Act for England and Wales. It looks in particular at thea shift in philosophy that the author claims has occurred with the proposals away from rights-focused principles to more utilitarian or outcome-focused principles. It gives examples of these changes and explores itstheir consequences.

## Background

The Draft Mental Health Bill 2004 was planned to be included in the Queen's Speech for the parliamentary session 2005/06, but this was later changed in the face of significant opposition to the Bill. Instead the government prepared amendments to the 1983 Mental Health Act, which are being debated in both Houses of Parliament [1] at the time when this article was written. These amendments [2] have to be understood in the context of new government priorities since 1997 [3] with the publication of the National Service Framework for Mental Health, the NHS Plan and the response to media coverage about psychiatric patients committing crimes. With the introduction of the Human Rights Act in 1998 (full implementation in 2000) a need for a new Mental Health Act was identified [4] to make current legislation comply with human rights legislation as well as bringing it up to date with 21^st ^century psychiatric practice, in particular community treatment.

## How to conduct an ethical analysis

Whilst many publications have addressed practical, political and financial aspects of the proposals as well as most recently, questions about the principles of the Bill [5], few have made a philosophically based ethical analysis of the proposed legislation. Any such analysis needs to look at the premises (the arguments) for the new legislation given by the government. These are, according to the Department of Health, the failure of community care, the need for new legislation and new community treatment realities [4]. It is then necessary to look at the proposed changes and the direction they take, before looking at the consequences. In a sound philosophical argument the consequences need to arise from their premises and need to be likely to achieve the stipulated aims [6]. In medical ethics we usually deal with different philosophical ideas, which sometimes have opposing directions as well as widely accepted medico-ethical principles such as non-maleficience, beneficience, rights and justice [6]. On the one hand there is utilitarian or consequentialist thinking, which tries to achieve the greatest happiness for the greatest numbers even though this may occasionally neglect the rights of individuals. On the other hand Kantian principles or rights focused approaches seek to protect individual rights and duties and are less concerned with outcome [7]. The present Mental Health Act is relatively rights-focused [8]. It endeavours to protect autonomous decision-making [7] with the help of concepts such as consent and capacity.

## The premises

Any ethical analysis starts with the premises put forward by the supporters of the proposals. The government states that care in the community has failed [4], although there is very little evidence to support this [10-14] and it is remarkable that the government's cornerstone of mental health care is nonetheless care in the community [4]. The available evidence points towards a success of care in the community. Shepard found in 1996 that the most disabled patients were still in long-stay psychiatric wards, but equally were the mostmore dissatisfied with their care compared to those living in the community [11]. Thornicroft showed in 1998 that a review of the evidence supports a community-oriented rather than a hospital-oriented approach; notably there is little difference between the various community mental health team models (such as assertive outreach or CMHT) [13]. Salize in an economic cost analysis stated that the cost of community care is only 43% of that of in-patient care [10]. In a comprehensive meta-analysis, Simmonds reviewed all of the available evidence on Community Mental Health Teams and came to the conclusion that CMHT treatment is superior to standard hospital treatment [12]. Whilst international comparisons remain difficult, the available evidence shows that care in the community does work on the whole, that it is both effective and cost effective, and improves patients' satisfaction with the services. Accordingly, patients' best interest may well be served best with care in the community, with hospitalisation a temporary measure to deal with acute episodes only.

The government focuses largely on risk, despite little evidence that risk assessments are reliable [15] or that risk has increased due to community treatments. The government claims that the present legislation is not adequate for modern 21^st ^century psychiatry, but there is little evidence that these problems could not be solved with amendments. The government is driven by media concerns about safety. The media implies that the mentally ill are potentially dangerous [16] despite lack of evidence that community care has increased violence by the mentally ill [17].

## Analysis of changes

The government's strategy for modernisation of Mental Health services hacomprises 3 elements, of which the new legal framework is one:

1. Increased investment in, and prioritisation of, mental health

2. Implementation of National Service Frameworks and the NHS plan in England and Wales

3. An up-to-date legal framework to "promotes patients' rights, protect their safety and protect the safety of the public" [18].

The third element is particularly important because it sets out the notion that the safety of the public is equally important as the promotion of patients' rights and the protection of the patients' safety. The other main reason cited by the government for the introduction of new legislation is the necessity for any Mental Health legislation to comply with the Human Rights Act 1998. The government specifically points out that compulsory treatment may be provided in the community as well as in hospital. It is claimed that this new focus on flexibility to meet individuals' needs will reduce stigma and social exclusion that can result from detention and treatment in hospital. In their own documentation the government argues that the proposed changes are designed to meet two primary needs:

a) to provide a legal structure for requiring mentally disordered people to submit to compulsory treatment without necessarily requiring them to be detained in hospital. This will allow their treatment outside hospital settings and the legislation governing it to comply more closely with the structures of modern mental health services. The new Bill will allegedly enable those services to be used more flexibly both for the benefit of mentally disordered people and for the protection of others from harm;

b) to bring the law more closely into line with modern human rights laws as defined by developing case law arising from the European Convention of Human Rights [3,19]. In particular, the Bill will require decisions to apply compulsion to mentally disorder people to be taken by an independent judicial body.

In order to achieve these objectives, the bill has proposed a number of key changes to the current 1983 Mental Health Act [2]:

1. Patients may be subject to community-based orders rather than detention in hospital. This means that more emphasis is placed on outcome because in the past it was often the non-compliance in the community that led patients to relapse and in rare cases become a danger to the public. From a utilitarian perspective this has to be welcomed. The problem is that treatment will have the potential to intrude upon a patient's privacy in an unprecedented way when involuntary treatment is not any longer limited to hospitals. This may infringe on the patient's right to privacy although it may very well be in his or her best interest.

2. The principles for involuntary admission set out in a code of practice do not apply to offenders who have been found by the courts to pose a risk of serious harm to others. According to the government the need to protect others from further harm committed by dangerous offenders means that safety considerations must be paramount in their clinical management. On the other hand, although the government promises that any unjustified intrusions by conditions applying to them will be avoided with special safeguards. The government goes on to suggest that people who have never committed any crime but are deemed potentially dangerous should be detainable against their will to protect the public despite warnings that up to 5000 people may have to be detained to prevent one homicide [15]. This suggestion reflects a clear shift away from liberal individualism towards utilitarian thinking and has attracted the greatest deal of criticism from many different mental health lobby groups [20]. It is a clear shift away from liberal individualism towards utilitarian thinking. The value of having a right to freedom has been overridden by the value of public safety. This means that the individual rights of the patient and potentially his or her best interest can be overruled by considerations of public safety, or in other words: the best interest, of the public. The problem with this proposal is not so much the shift itself, since that is a legitimate political decision, but the practicalities of it. The most reliable predictor of violence in a person is previous violence, which means that to predict violence in a person who has never been violent is at best unreliable and contradicts all evidence that has been gathered on the prediction of violence. Thus it is possible that people can loose their right to freedom to the best interest of the public without ever having been a danger. It is that potential that should concern even supporters of the utilitarianist model, because the consequence of too many people who are wrongfully detained could be a collapse of trust in the system, which would be a very unsatisfactory outcome.

3. The amendments introduce new safeguards for informal patients with long-term incapacity who cannot consent to treatment but are not resisting it and are therefore de facto detained. This strengthens the rights of these patients, who were previously detained in their best interest, but without legal safeguards to check whether the responsible clinicians professionals looking after them were indeed acting in their best interest. This is therefore not a shift in principle, but a legal strengthening of safeguard by legal means.

4. A very broad single definition of a mental disorder is introduced in the new Bill, which means that all patients will be considered against the same set of conditions. There will therefore be no need to distinguish between mental impairment, mental disorder and psychopathic disorder, making detention for non-psychiatric reasons much more likely. This again supports the tendency of the amendments to shift towards more utilitarian thinking. The fact that detention will not any longer be possible to prevent deterioration of health but only when there is risk appears to make detention more difficult, but in fact it removes the very criterion that has the patient's best interest at heart, in favour of a broader best interest of society and the management of risk.

5. The new Mental Health Tribunals (MHRT) are not required to contain a psychiatrist. So-called general members can be recruited from a pool of mental health professionals with a defined minimum number of years experience in mental health, this will include psychologists, social workers and psychiatric nurses. Research suggests that this may include professionals with a more potentially hostile view (see discussion below) towards formal detention as long as they are not party to the process [21,22]. The consequences of such a shift are not yet clear, but may shift the emphasis of tribunals' decisions at least temporarily to an approach that favours individual rights. The Tribunals will have order making powers to reduce the time before a patient is seen by the MHRT. This will increase patients' rights and make current legislation comparable with article 3 of the Human Rights Act 1998.

An analysis of the proposed changes shows that some changes are moving into a more utilitarian or consequential direction whereas others move into a more rights focused direction. The introduction of community orders to enable compulsory treatment in the community is a clear move towards more utilitarian approaches. So is the broad single definition of mental disorder, because they both favour public safety considerations over individual rights. The detention of patients with severe personality disorders clearly points towards a more utilitarian thinking even though the evidence as to whether this is going to increase public safety [15] is very ambiguous. In fact, the priority of safety is such that the government, in its own words, states, "safety considerations must be paramount in clinical management" [4]. Safeguards for patients with long-term incapacity move towards a more rights focused approach, as do tribunals being held within a tight time frame. Mental health tribunals without psychiatrists on the panel, as will be commonplace under the new proposed legislation, may skew panel decisions against compulsory admissions [21,22], which may temporarily strengthen individual rights. This can be seen as utilitarian or rights focused, but the consequences are still very unclear. In summary, there is a clear ethical shift towards more consequentialist thinking, or in other words: outcome oriented thinking, away from rights focused approaches.

## The consequences

When analysing the consequences, the research into community orders comes from the United States and Australia, is of questionable generalisability for the UK and renders ambiguous results [23,24]. Community orders exist in a third of countries in the European Union [25], but very little research exists that would allow an analysis of their effectiveness in the European context. It is very unlikely that the proposed legislation will reduce violence by mentally ill offenders [26]. Society is likely to feel equally unsafe because of media coverage regardless of whether this new focus is implemented or not. Psychiatrists are pressurised into a policing role which many detest [27] and which may have an adverse effect on recruitment. There is a high likelihood of increased stigmatisation of mental illness due to the legislation rather than the intended reduction of stigma.

## Discussion

In summary, the proposed legislation changes mean an ethical shift away from rights focused approaches to more consequentialist thinking [Table 1]. The changes are politically legitimate, but from an ethical point of view any shift away from rights focused thinking would only be desirable if there were overwhelming benefits to society. Any ethical analysis needs to be based on the overall premises (is the driving force for change justifiable) and consequences (will things improve for patient and the public) need to logically follow from the premises. Currently the consequences do not follow logically from their premises. Therefore the proposed changes to the Mental Health Act 1983 establish questionable consequences, which do not follow from the underlying premises. The premises themselves are little supported by evidence. This is an ethically unacceptable approach and should be resisted on ethical grounds until premises and consequences can be more reliably analysed.

**Table 1:** Overview of proposed changes and their ethical assessment

**Proposed legislation changes**	**Utilitarian**	**++**	**+**	**0**	**-**	**--**	**Rights-focused**
Community supervision		x					
Broad definition of mental disorder		x					
Detention of DSPD		x					
More general tribunal members				x			
Communication amongst services		x					
Overall assessment			x				

## Competing interests

The author(s) declare that they have no competing interests.
